# Nonfamilial Juvenile Polyposis Syndrome with Exon 5 Novel Mutation in SMAD 4 Gene

**DOI:** 10.1155/2017/5321860

**Published:** 2017-03-27

**Authors:** Amna Ahmed, Badr Alsaleem

**Affiliations:** Pediatric Gastroenterology and Hepatology Division, Children's Hospital, King Fahad Medical City, Riyadh, Saudi Arabia

## Abstract

Juvenile polyposis syndrome (JPS) is a rare autosomal dominant hereditary disorder, characterized by multiple juvenile polyps in the gastrointestinal tract and an increased risk of colorectal cancer. JPS is most frequently caused by mutations in the SMAD4 or BMPR1A genes. Herein, we report a child with juvenile polyposis syndrome (JPS) with a novel mutation in the SMAD4 gene. An 8-year-old boy presented with recurrent rectal bleeding and was found to have multiple polyps in the entire colon. The histology of the resected polyps was consistent with juvenile polyps. Subsequent genetic screening revealed a novel mutation in SMAD4, exon 5 (p.Ser144Stop). To the best of our knowledge, this mutation has not been reported before. Offering genotypic diagnosis for patients with JPS is an important step for strategic plan of management.

## 1. Introduction

Juvenile polyposis syndrome (JPS) is a rare autosomal dominant hereditary disorder with an estimated incidence of 1 in 100,000–160,000 individuals. It is characterized by multiple distinct juvenile polyps in the gastrointestinal tract and an increased risk of colorectal cancer [1]. This disorder is most frequently caused by mutations in the SMAD4 or BMPR1A genes, but together these genes accounts for only 40% of cases [2].

## 2. Case Report

**Figure 1:**
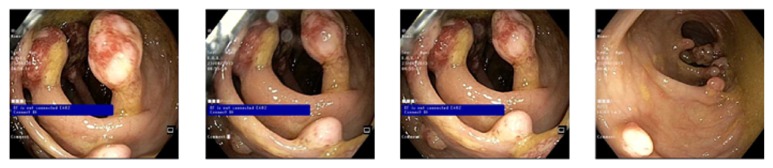
Multiple pedunculated and sessile, nonbleeding polyps were found in the entire colon more and larger in the right colon. They are >80 in number with variable sizes (ranging from few millimeter to 3 cm).

**Figure 2:**
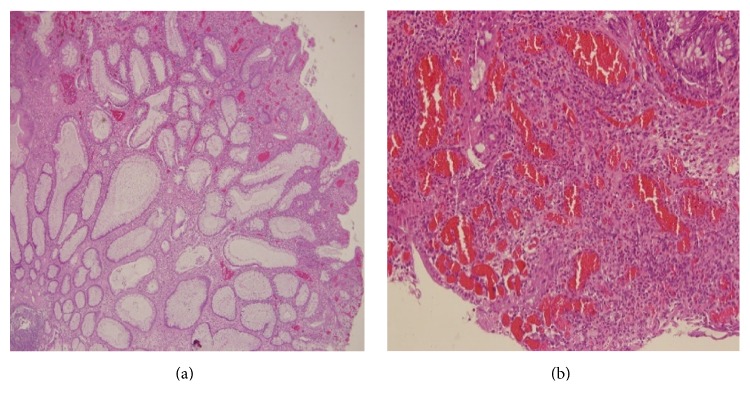
Edematous lamina propria, cystically dilated glands (a) with inflammatory cells (b) in resected tissue obtained from colonoscopic polypectomy (H&E stain, ×40).

**Figure 3:**
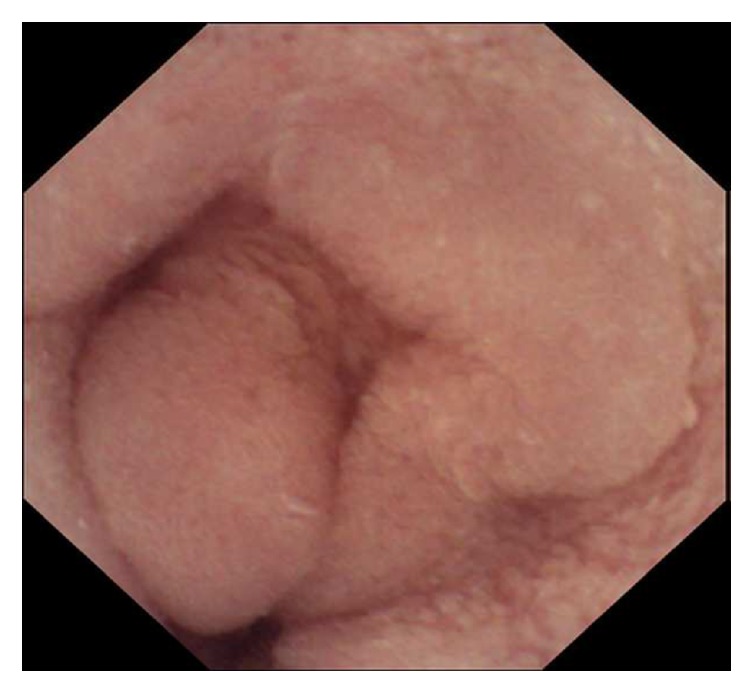
Video capsule endoscopy (VCE): One image from small intestine: No polyps seen.

An 8-year-old Saudi boy presented to the Gastroenterology service, with a 3-year history of recurrent rectal bleeding, generalized abdominal pain, loose motions, and fatigability. The patient is product of a consanguineous marriage and family history is unremarkable for gastrointestinal (GI) bleeding or GI malignancies. Apart from pallor, physical examination was unremarkable. Initial laboratory work up showed features of iron deficiency anemia. Esophagogastroduodenoscopy (EGD) was unremarkable. However, colonoscopy revealed dozens of pedunculated and sessile polyps of different sizes (range of 10–30 mm), distributed along the entire length of the colon (Figure 1). The histological analysis of the polyps showed hamartomatous features that were consistent with juvenile polyposis (Figure 2). Video capsule endoscopy (VCE) excluded small intestinal involvement (Figure 3). The diagnosis of JPS was made, based on endoscopic and clinicopathological findings. After obtaining a written and informed consent, genetic test was done, detecting a previously nonreported, heterozygous mutation in SMAD4: sequence defined as c.431C>G and predicted to result in premature protein termination (p.Ser144Stop), in exon 5. However, the patient's biological members genetic screening was unremarkable.

Attempts for endoscopic colonic polyp clearance were made, but this had been unsuccessful due to large polyps burden. Given the worsening of gastrointestinal bleeding and iron deficiency anemia and avoiding other complications such as intussusception and colorectal cancer, total colectomy was done. Long term follow-up was planned by conducting surveillance with upper endoscopy every 1 to 2 years and small bowel evaluation every 2 to 3 years.

## 3. Discussion

The case describes an 8-year-old male, proved to have JPS, and the genetic study detected a nonfamilial, novel spontaneous mutation in SMAD4 in exon 5.

Approximately, 20–50% of patients with PJS have a family history, while about 25–50% of patients have new mutations [3].

The most common mutation in SMAD4 is a 4-base deletion at exon 9 [4]. Other reported novel gene mutation in SMAD4 was found in exon 10, in a family with familial juvenile polyposis syndrome (FJP) [5].

Common presenting symptoms of JPS include rectal bleeding, anemia, abdominal pain, and less commonly rectal prolapse of polyps [6].

If JPS diagnosis is made, the entire gastrointestinal tract should be examined endoscopically or radiologically for detecting polyps. Video capsule (VCE) is one of modalities used for polyps detection [7]. In our case VCE was done (preceded by abdominal computerized tomography scan) and it was unremarkable for small intestinal polyps. Macroscopically, juvenile polyps vary in size from 5 to 50 mm, typically having a spherical, lobulated, and pedunculated appearance, with surface erosion. Microscopic features include edematous lamina propria with inflammatory cells and cystically dilated glands [1].

SMAD4 gene mutations predispose to a more aggressive gastrointestinal phenotype with increased risk for gastric and colorectal cancer, compared to other mutations such as the BMPR1A. Patients with JPS should be subjected to yearly endoscopic clearance until they are deemed polyp-free [8]. Thus, genotypic diagnosis for children affected by JPS is an important step in the management. Once the gene mutation is identified, screening for at risk family members will be planned for and the future surveillance strategic plan for the gene carriers will be determined.

Prophylactic colectomy should be considered in those with large colonic polyps burden in whom endoscopic clearance is not possible, in patients with severe gastrointestinal bleeding or diarrhea, and, lastly, if there is a strong family history of colorectal cancer [9]. Hence, prophylactic total colectomy was done for our patient.

## 4. Conclusions 

To the best of our knowledge, this SMAD 4 gene mutation, in patients with JPS, was not described before.

Offering genotypic diagnosis for patients affected by JPS is an important step in the strategic plan of management.
