# Corrigendum to “Nonfamilial Juvenile Polyposis Syndrome with Exon 5 Novel Mutation in SMAD 4 Gene”

**DOI:** 10.1155/2017/9861278

**Published:** 2017-11-14

**Authors:** Amna Ahmed, Badr Alsaleem

**Affiliations:** ^1^Pediatric Gastroenterology and Hepatology Division, Children's Hospital, King Fahad Medical City, Riyadh, Saudi Arabia; ^2^Faculty of Medicine, King Saud bin Abdulaziz University for Health Sciences, Riyadh, Saudi Arabia

In the article titled “Nonfamilial Juvenile Polyposis Syndrome with Exon 5 Novel Mutation in SMAD 4 Gene” [1], there was a missing affiliation for the second author. The corrected affiliation list is shown above.
